# Addition of Aripiprazole to the Clozapine May Be Useful in Reducing Anxiety in Treatment-Resistant Schizophrenia

**DOI:** 10.1155/2011/846489

**Published:** 2011-09-14

**Authors:** Aleksandar Chanachev, Nicolas Ansermot, Séverine Crettol Wavre, Ute Nowotka, Maria-Eleni Stamatopoulou, Philippe Conus, Chin B. Eap

**Affiliations:** ^1^Department of Psychiatry, Centre Hospitalier Universitaire Vaudois, Section Eugene Minkowski, Site de Cery, 1008 Prilly, Switzerland; ^2^Unité de Biochimie et Psychopharmacologie Clinique, Centre de Neurosciences Psychiatriques, Site de Cery, 1008 Prilly, Switzerland; ^3^Department of Psychiatry, Centre Hospitalier Universitaire Vaudois, Psychiatrie de Liason-Urgences et Crise, 1011 Lausanne, Switzerland

## Abstract

There exist many case reports and studies on the antipsychotic augmentation by aripirazole in partial responders to clozapine, the most seem to be finding a slight difference in the PANSS and CGI scores after the aripirazole addition. The results of our report are compatible with those of other studies but, we have found a considerable antianxiety action in both of the cases. The 5HT1A agonism of aripirazole could be hypothesized as mechanism contributing to this effect.

## 1. Introduction

Clozapine is the drug of choice in treatment-resistant schizophrenia, but 40–70% of clozapine-treated patients continue to demonstrate suboptimal clinical response [1–4]. 

Various augmentation strategies have been tested, including the use of other atypical antipsychotics, but no clear recommendations can presently be proposed [5–10]. 

Augmentation with aripiprazole has been documented in case reports [11], in open trials [5, 12], and in a randomized controlled study [13].

In this paper, we report on 2 cases in which augmentation with aripiprazole had a beneficial impact on anxiety.

## 2. Case Presentation

### 2.1. Case Report 1

Ms. A, a 40-year-old woman diagnosed with a residual schizophrenia [14] was admitted following an exacerbation of psychotic symptoms with a predominance of anxiety despite 700 mg/d of clozapine for two years. The clinical scores and the trough plasma concentrations of clozapine and norclozapine at admission were CGI : 5; total PANSS: 123; positive: 17/49; negative: 18/49; excited component: 12/35; general: 39/112; Hamilton-anxiety: 14/56; clozapine: 896 ng/mL; norclozapine: 551 ng/mL, respectively (clozapine therapeutic range: 350–600 ng/mL [15–17]). Because of the risks of seizures, the dose was reduced to 500 mg/d, and aripiprazole (10 mg/d) was added. Clozapine and norclozapine plasma concentrations measured after 10 days were decreased according to the reduction of the dose (615 ng/mL and 478 ng/mL, resp.). The aripiprazole plasma concentration after 10 days was 282 ng/mL. Following a clinical reduction of the anxiety, the patient was discharged from the hospital three weeks after the addition of aripiprazole.

A followup over 6 months did not reveal any change in the CGI and PANSS scores (at 6 months: CGI : 5; total PANSS: 125; positive: 18/49; negative: 23/49; excited component 10/35; general: 38/112). On the other hand, the Hamilton-anxiety score diminished progressively to 12, 7, and 8 after one, three, and 6 months, respectively. Clozapine and norclozapine plasma levels were stable over this period (at the sixth month: 608 ng/mL, 443 ng/mL, and 75 kg, resp.), and the comedications (clorazepate 20 mg/d, valsartane 40 mg/d, zopiclone 7,5 mg/d, and tamsulosine 0.4 mg/d) were not modified. No reports are describing an impact on anxiety by the antihypertensive comedication by valsartane (an angiotensin II receptor antagonist) and tamsulosine (peripheral *α*1-antagonist). There is no significant pharmacokinetic or pharmacodynamic interactions of that comedication and the antipsychotic/anxiolytic treatments.

### 2.2. Case Report 2

Mr. L, a 48-year-old man with a diagnosis of residual schizophrenia [14] treated for many years with clozapine 500 mg/d was admitted because of the worsening of his anxiety. The clinical scores and the trough plasma concentrations of clozapine and norclozapine were: CGI : 4; total PANSS: 88; positive: 20/49; negative 25/49; excited component: 9/35; general 40/112. Hamilton-anxiety: 24/56; clozapine: 594 ng/mL; norclozapine: 470 ng/mL, respectively. Aripiprazole 10 mg/d was added to clozapine (dose unchanged) and lorazepam (1 mg/d) was discontinued. The patient was discharged three weeks after the aripiprazole addition.

A followup over 3 months did not reveal any change in the CGI and PANSS scores (at 3 months: CGI : 4; total PANSS: 73; positive: 20/49; negative: 25/49; excited component: 7/35; general: 40/112), while the Hamilton-anxiety score diminished progressively to 19 and 15 after one and three months, respectively. The clozapine and norclozapine plasma concentrations at three months were 431 ng/mL and 343 ng/mL, respectively.

## 3. Discussion

In the present paper the augmentation did not result in a reduction of psychotic symptoms despite a treatment period of 3 to 6 months, which is in agreement with previous reports [5, 10–13]. Because a therapeutic window has been demonstrated for clozapine [17, 18], it is important to mention that therapeutic blood levels of clozapine were maintained during the whole observation period. An important reduction of anxiety was clinically observed in both cases, with a marked improvement of psychosocial functioning observed, which allowed a change of residential institution of both cases, three and six months after the discharge from the hospital, for a residential stay in more open environment with less psychosocial accompanying measures. 

It has been suggested that in anxiety disorders, the adjunction of atypical antipsychotics to the current SSRI and/or benzodiazepine treatment could, through the modulation of the dopaminergic system, be beneficial but the data are not conclusive [19, 20].

The agonist action of aripiprazole on the 5HT1A receptors could eventually contribute to the antianxiety action that we have observed [21].

However, considering the present observations could be due to external factors or to the natural evolution of the illness, a randomized controlled study is required to evaluate the efficacy of the clozapine-aripiprazole combination in cases of treatment-resistant schizophrenia with predominance of anxiety. Moreover, the anxiety observed in Case 1 could have been in part attributable to psychotoxic effects due to the high plasma concentration at the begining of the followup [22].
